# Scenario-Driven Rapid Testing for Top Pathogens in Pediatric Respiratory Infections: Clinical and Economic Value from Emergency Triage to Precision Anti-Infective Management in the PICU

**DOI:** 10.3390/pathogens15060628

**Published:** 2026-06-12

**Authors:** Jiahui Chen, Huaying Wang, Ying Li, Yuyi Xiao, Yi Yan, Yifei Zhang, Xiaoxia Lu

**Affiliations:** 1 Sanya Institute of Hunan University of Science and Technology, Hunan University of Science and Technology, Sanya 572024, China; jiahuichen994@gmail.com; 2 Department of Respiratory Medicine, Wuhan Children’s Hospital, Tongji Medical College, Huazhong University of Science and Technology, Wuhan 430014, China; 18371929121@163.com (H.W.); elise_lii@163.com (Y.L.); xyy15572223229@163.com (Y.X.); 3 Pediatric Respiratory Disease Laboratory, Institute of Maternal and Child Health, Wuhan Children’s Hospital, Tongji Medical College, Huazhong University of Science and Technology, Wuhan 430014, China

**Keywords:** diagnostic stewardship, multiplex PCR, pediatric respiratory infections, rapid diagnostics, top pathogens

## Abstract

Pediatric respiratory infections remain among the leading causes of emergency department visits, hospitalization and pediatric intensive care unit (PICU) admission. Although most acute respiratory infections in children are viral, clinical manifestations overlap substantially among viral, bacterial and atypical pathogens, creating diagnostic uncertainty and promoting empirical antimicrobial use. Rapid antigen tests, nucleic acid amplification tests, multiplex respiratory panels and metagenomic sequencing have expanded the ability to detect pathogens within clinically actionable timeframes. However, evidence from pediatric emergency trials indicates that rapid pathogen detection alone does not necessarily reduce antibiotic prescribing or healthcare costs. These findings suggest that the value of rapid diagnostics depends less on analytical breadth than on whether testing is applied to the right child, in the right clinical scenario and within a predefined decision pathway. This narrative review reorganizes the evidence around a scenario-driven top-pathogen framework. Top pathogens are defined as organisms that, in a specific age group, syndrome, season or care setting, have high prevalence, severe disease potential, transmissibility, treatment implications, antimicrobial resistance relevance or infection-control value. We discuss how top-pathogen testing should differ across emergency triage, inpatient ward management, severe pneumonia, PICU care, hospital-acquired pneumonia, ventilator-associated pneumonia and outbreak settings. We further examine the economic mechanisms through which rapid testing may generate value, including reduced unnecessary antibiotics, timely antiviral therapy, optimized isolation, shorter length of stay, reduced repeated testing and prevention of healthcare-associated transmission. Finally, we propose implementation principles centered on diagnostic stewardship, antimicrobial stewardship, local epidemiology and real-world cost-effectiveness evaluation. A scenario-driven top-pathogen strategy may provide a practical bridge between broad syndromic testing and precision infectious disease management in children.

## 1. Introduction

Acute respiratory infections are among the most frequent reasons for pediatric emergency department attendance, hospitalization and PICU admission. Children with fever, cough, wheezing, dyspnea, hypoxemia or radiographic pneumonia often present with overlapping clinical phenotypes despite different etiologies [1]. Respiratory syncytial virus (RSV), influenza virus, rhinovirus, human metapneumovirus (hMPV), adenovirus, severe acute respiratory syndrome coronavirus 2 (SARS-CoV-2), *Mycoplasma pneumoniae*, *Bordetella pertussis* (*B. pertussis*), *Streptococcus pneumoniae* (*S. pneumoniae*), *Haemophilus influenzae* (*H. influenzae*) and *Staphylococcus aureus* (*S. aureus*) may all cause pediatric respiratory disease, yet the treatment and infection-control implications differ considerably [2,3,4].

Traditional diagnostic pathways remain imperfect. Bacterial culture remains clinically important but is often too slow for early therapeutic decision-making, generally requiring at least 24–48 h for preliminary organism identification or definitive results, and even longer when antimicrobial susceptibility testing is required, when slow-growing or fastidious organisms are involved, or when specimen quality is suboptimal or antibiotics have been administered before sampling [5]. Single-pathogen tests are useful during specific seasons or outbreaks but do not adequately address co-circulating pathogens. Clinical features and inflammatory biomarkers help stratify risk, but they cannot reliably distinguish viral, bacterial and atypical etiologies in many children [6]. This diagnostic uncertainty often leads to empirical antibiotic prescribing, delayed antiviral therapy, inefficient isolation decisions and avoidable healthcare utilization. Among these consequences, unnecessary or prolonged empirical broad-spectrum antibiotic exposure is particularly important because it may promote antimicrobial resistance and selection pressure, while also contributing to drug-related adverse events, nephrotoxicity associated with selected agents and the disruption of the developing pediatric microbiome [7,8]. These potential harms further support the need for rapid, scenario-driven diagnostic strategies that return clinically actionable results within early decision-making timeframes.

Rapid molecular diagnostics have changed the technical possibilities of pathogen identification. Modern nucleic acid amplification tests and multiplex polymerase chain reaction (PCR) panels can detect multiple respiratory viruses, atypical bacteria and, in some platforms, bacterial pathogens or resistance markers within minutes to hours [9,10]. Pediatric community-acquired pneumonia (CAP) guidelines have recognized that rapid testing for influenza and other respiratory viruses may reduce additional diagnostic procedures and antibiotic use when bacterial co-infection is not suspected, and that *Mycoplasma* testing may guide antimicrobial selection in compatible clinical syndromes [11]. Population-based CAP studies further show that respiratory viruses are frequently detected in hospitalized children, while atypical pathogens such as *M. pneumoniae* become more relevant in older children [12].

Nevertheless, rapid detection has not produced uniformly positive clinical effects. Pediatric emergency department randomized trials have shown that rapid respiratory pathogen testing does not automatically reduce antibiotic prescribing [13,14]. A systematic review and meta-analysis of randomized trials also found moderate-quality evidence that rapid point-of-care respiratory pathogen testing does not reduce antibiotic prescription rates overall [15]. In contrast, the OPTIPAC randomized trial in pediatric CAP demonstrated that rapid multiplex PCR improved the appropriateness of initial antimicrobial management, mainly by reducing unnecessary antibiotics in viral pneumonia [16,17].

These apparently divergent findings point to a central principle: rapid diagnostics should not be evaluated as stand-alone laboratory technologies. Their clinical and economic value depends on scenario selection, turnaround time, pathogen actionability, clinician response and integration with diagnostic and antimicrobial stewardship. Therefore, this review proposes a scenario-driven top-pathogen approach for pediatric respiratory infections. The goal is to shift from the question ‘How many pathogens can be detected?’ to the more clinically relevant question of ‘Which pathogens should be rapidly detected in this child, in this setting, to change management and reduce clinical and economic burden?’

This article was designed as a narrative review. Relevant literature was identified primarily through PubMed searches and was supplemented by manual screening of reference lists from key articles, as well as relevant guideline and policy documents from professional organizations and public-health agencies. The search focused mainly on publications from January 2010 to May 2026, while earlier landmark guidelines or foundational studies were included when they remained clinically relevant. Search terms included relevant MeSH terms and free-text terms, including “pediatric respiratory infection”, “children”, “rapid diagnostics”, “multiplex PCR”, “respiratory pathogen panel”, “molecular testing”, “diagnostic stewardship”, “antimicrobial stewardship”, “emergency department”, “pediatric intensive care unit”, “severe pneumonia”, “community-acquired pneumonia”, “hospital-acquired pneumonia”, “ventilator-associated pneumonia”, “cost-effectiveness” and “health economics”. We prioritized pediatric randomized trials, observational studies, systematic reviews, clinical guidelines, diagnostic stewardship studies, antimicrobial stewardship studies and health-economic evaluations relevant to respiratory pathogen testing. Adult or mixed ICU studies were considered only when pediatric-specific evidence was limited and when the findings were relevant to PICU, hospital-acquired pneumonia or ventilator-associated pneumonia scenarios. Because this was a narrative review, no formal meta-analysis, risk-of-bias assessment or PRISMA-based study selection process was performed. Studies were not prioritized when they were unrelated to pediatric respiratory infections, lacked relevance to diagnostic decision-making, antimicrobial stewardship or health-economic outcomes, or did not provide sufficient clinical or methodological detail.

## 2. Conceptual Framework: From Broad Pathogen Detection to Scenario-Driven Top-Pathogen Testing

### 2.1. Defining Scenario-Driven Top-Pathogen Testing

In this review, a top pathogen is defined as a pathogen that should be prioritized for rapid detection in a defined pediatric respiratory infection scenario because of high local prevalence, severe disease potential, transmissibility, the availability of targeted therapy, implications for antibiotic de-escalation, relevance to antimicrobial resistance or importance for isolation and outbreak control. This definition is deliberately broader than a prevalence ranking. A pathogen can be common but clinically non-actionable in a particular setting, whereas a less frequent pathogen may be a priority target if early recognition changes treatment or infection-control actions [18].

Operationally, hospitals can translate this definition into a scenario-specific prioritization process by first defining the clinical scenario and then assessing candidate pathogens across several practical dimensions: local prevalence or seasonal activity, potential to cause severe disease or clinical deterioration, clinical actionability, transmissibility and infection-control consequences, and antimicrobial resistance or resistance-marker relevance. When these dimensions conflict, pathogens should not be prioritized solely according to detection frequency. Instead, priority should be given to pathogens for which early detection is most likely to change treatment, antimicrobial escalation or de-escalation, isolation, cohorting or public-health actions. This process is intended as a flexible decision aid rather than a fixed scoring rule. Accordingly, top-pathogen lists should not be treated as universal or static. They should be periodically reviewed and updated using local laboratory surveillance, age- and ward-specific pathogen distribution, seasonal trends, vaccination coverage, antimicrobial resistance patterns, outbreak reports, stewardship audit data and available diagnostic resources, because priority pathogens may differ across countries, epidemic seasons and levels of hospital referral.

For example, RSV is a top pathogen in infants with bronchiolitis because of its burden, association with oxygen requirement, seasonal clustering and relevance to cohorting. Influenza virus is a top pathogen during seasonal circulation. In temperate regions, influenza activity generally increases in autumn and winter and often peaks in winter, although timing varies by region and year. In this context, rapid diagnosis may support timely antiviral therapy, reduce unnecessary antibacterial exposure and guide isolation [19]. *B. pertussis* is a top pathogen in young infants, prolonged paroxysmal cough and household clusters because detection triggers targeted macrolide therapy, contact tracing and public-health measures [20]. In PICU or ventilated patients, *S. aureus*, *Pseudomonas aeruginosa* (*P. aeruginosa*), *Klebsiella pneumoniae* (*K. pneumoniae*), *Acinetobacter baumannii* (*A. baumannii*) and resistance markers become top targets because delayed appropriate therapy and unnecessary broad-spectrum exposure both carry major consequences.

This logic is consistent with global pathogen-prioritization concepts. The 2024 World Health Organization Bacterial Priority Pathogens List emphasizes prioritization based on burden, transmissibility, treatability, prevention options and antimicrobial resistance trends, and includes resistant Gram-negative bacteria, *P. aeruginosa* and *S. aureus* among important priority groups [21]. The same principles can be adapted locally in pediatric hospitals to design age-, season- and severity-specific respiratory testing algorithms (see Table 1).

Given the geographical and epidemiological heterogeneity of the evidence base, the proposed framework should be interpreted as a locally adaptable decision tool rather than a universal pathogen list. Studies discussed in this review were derived from diverse regions and healthcare systems, including North America, Europe, Asia and international policy documents. Therefore, local implementation should account for regional seasonality, vaccination coverage, circulating pathogens, resistance ecology, diagnostic availability and healthcare-resource constraints.

### 2.2. Why Broad Detection Alone Is Insufficient

The appeal of broad multiplex testing is intuitive: more targets, faster results and higher diagnostic yield. However, increased detection does not necessarily equal improved care [22]. The central challenge is diagnostic causality: molecular panels identify microbial nucleic acid, but they do not by themselves prove active disease or explain the child’s current clinical syndrome. Positive results must be interpreted in relation to sampling site, illness timing, pre-test probability, disease severity, biomarkers, radiology and the expected management consequence. Negative results may falsely reassure clinicians when the sample is inadequate, the pathogen is outside the panel, pathogen burden is low or disease is primarily located in the lower airway while only an upper-airway sample is tested. When a broad panel provides a microbiological label that does not explain the current illness, pathogen overdiagnosis may occur. This may increase interpretive burden, reinforce uncertainty and, in some cases, contribute to unnecessary treatment escalation or continued antibiotic exposure. Children are especially vulnerable to interpretive complexity. Rhinovirus and adenovirus may be detected during prolonged shedding or after recent infection, whereas *S. pneumoniae* and *H. influenzae* may represent upper-airway colonization rather than invasive or lower-airway disease. Multiple viruses may be detected during or after sequential infections. In addition, upper-airway detection may be difficult to interpret when the clinically relevant disease process is located in the lower airway. These issues are particularly important for broad multiplex panels, because overinterpretation of viral or bacterial detections may lead to unnecessary antimicrobial escalation, reluctance to de-escalate empirical antibiotics, or increased antibiotic pressure despite stewardship-oriented testing. In the absence of interpretive guidance, co-detection can even reinforce clinician uncertainty and promote continued antibiotic use [10].

The pediatric randomized evidence reinforces this point. In one ED trial, the knowledge of rapid respiratory pathogen results within approximately one hour did not reduce antibiotic prescribing among children with influenza-like illness, although antiviral prescribing became more judicious [13]. In another trial, multiplex PCR point-of-care testing for 18 viruses and three bacteria did not reduce antibiotic use, diagnostic testing or costs among 1243 acutely ill children [14]. These studies do not argue against rapid testing; rather, they show that test availability alone is insufficient. Clinical value should therefore be understood as conditional rather than automatic: it emerges when testing is targeted to patients in whom results can change a specific decision and when results are interpreted within the full clinical context.

### 2.3. Matching Diagnostic Platforms to Clinical Questions

A scenario-driven strategy requires matching the diagnostic platform to the clinical question. Rapid antigen tests are fast, low-cost and operationally simple, making them useful for selected pathogens such as influenza, RSV and SARS-CoV-2 when a positive result is actionable. Their limitations include lower sensitivity than nucleic acid amplification tests and weaker performance when viral load is low or sampling is suboptimal.

Singleplex PCR or small multiplex PCR assays are appropriate when the likely pathogens are limited and highly actionable. Examples include influenza/RSV/SARS-CoV-2 testing during respiratory virus season, targeted *M. pneumoniae* testing in school-aged pneumonia and *B. pertussis* testing in infants or prolonged cough syndromes [23]. Small panels may be particularly useful in emergency departments because they balance speed, interpretability and cost [24].

Broad respiratory panels are better reserved for children in whom broad results are likely to affect management: hospitalized patients, severe pneumonia, immunocompromised children, PICU admission, suspected mixed infection, infection-control events or failure of empirical therapy. Pneumonia or HAP/VAP panels with bacterial and resistance targets are most useful when lower-airway disease and antimicrobial optimization are central [25]. Metagenomic or metatranscriptomic sequencing is best positioned as an escalation strategy for severe unexplained disease, immunocompromised hosts, negative conventional testing or suspected unusual pathogens [26]. The 2025 WHO diagnostic landscape report highlights continuing needs for rapid tests that distinguish bacterial from viral infection, identify pathogens and detect susceptibility or resistance (Table 2) [27].

### 2.4. A Scenario-Driven Testing Model

In practice, scenario assignment should occur before the etiology is known and should be based on information available at the initial encounter. Pediatric respiratory presentations frequently overlap, and bronchiolitis, pneumonia, viral wheezing, asthma exacerbation, bacterial co-infection and mixed infection may be difficult to distinguish at presentation. Therefore, clinicians should first identify the dominant clinical presentation and then refine the testing strategy according to age, oxygen requirement, disease severity, radiographic findings, immune status, hospitalization or ventilation status, seasonality, local epidemiology and outbreak exposure. When clinical categories overlap, the testing pathway should generally follow the highest-risk or most clinically actionable scenario, while avoiding broad low-value testing in children whose management is unlikely to change. Table 2 provides a practical scenario-classification approach for assigning children to initial testing pathways before etiologic confirmation.

Based on this initial classification, the proposed model has six linked components: initial presentation, key decision points, scenario classification, testing pathway selection, actionable clinical decisions and real-world feedback. First, clinicians assess the child’s initial presentation before the etiology is known, including fever, cough, wheeze, dyspnea, hypoxemia and overlapping respiratory features. Second, key decision points, including age, oxygen requirement, disease severity, immune status, seasonality, local epidemiology, biomarkers and radiology, are used to assign the child to the most relevant testing scenario. Third, children are classified into practical scenarios such as bronchiolitis, CAP, viral wheezing/asthma exacerbation, suspected mixed infection, PICU severe pneumonia, HAP/VAP or outbreak settings. Fourth, testing pathway is selected according to diagnostic breadth and turnaround-time requirements, ranging from targeted testing or small respiratory panels to broad respiratory panels, pneumonia/HAP/VAP panels or mNGS/metatranscriptomics in selected cases. Fifth, results are linked to specific actions, including antiviral treatment, antibiotic withholding, escalation or de-escalation, isolation, cohorting and admission or PICU management. Finally, real-world outcomes are monitored to update local top-pathogen lists, testing criteria and stewardship pathways. This approach does not reject broad multiplex testing. Instead, it reserves broad testing for high-complexity scenarios while encouraging targeted or small-panel strategies for high-volume, lower-risk settings. It also makes explicit that rapid testing is not the endpoint. The endpoint is better clinical decision-making.

## 3. Clinical Utility Across the Pediatric Respiratory Care Continuum

### 3.1. Emergency Department Triage: Rapid Decisions Within a Narrow Time Window

The emergency department is a high-volume, time-constrained setting in which rapid testing can influence triage, antiviral use, antibiotic decisions, isolation, observation, admission and PICU transfer [32]. The relevant question is not simply whether a pathogen is present, but whether the result will return before the clinician has finalized key decisions [33,34]. A result that arrives after antibiotics, imaging and disposition have already been determined has less immediate clinical value.

For otherwise healthy children with mild upper respiratory symptoms, broad respiratory panels are rarely necessary. Targeted testing for influenza, RSV or SARS-CoV-2 may be appropriate during seasonal circulation, when results affect antiviral treatment, infection-control advice, household risk or school guidance. For infants with bronchiolitis, RSV, rhinovirus, hMPV, parainfluenza virus (PIV) and influenza virus are important targets, but the purpose should be explicit: cohorting, avoidance of unnecessary antibiotics, estimation of oxygen or admission risk, or management of high-risk infants. For children with suspected CAP requiring ED evaluation, the target list should broaden to include respiratory viruses, *M. pneumoniae* and *B. pertussis*, and sometimes bacterial targets when disease is severe.

The evidence suggests that unselected rapid testing in children with broad acute respiratory symptoms is unlikely to transform antibiotic prescribing by itself [13,14,15]. However, syndrome-specific deployment appears more promising. In the OPTIPAC trial, rapid multiplex PCR in children with CAP improved the appropriateness of initial antimicrobial management, with benefit driven largely by avoiding antibiotics in viral pneumonia [16]. Therefore, ED testing should be designed around narrow decision windows and clearly defined clinical syndromes rather than applied uniformly to every child with respiratory symptoms.

### 3.2. Inpatient Ward Management: Antibiotic De-Escalation and Isolation Optimization

In hospitalized children, rapid testing has a different role. The primary value may be ongoing treatment optimization rather than immediate triage. Results can inform antibiotic continuation or de-escalation, antiviral treatment, isolation duration, cohorting, additional diagnostic work-up and anticipated length of stay [35]. Viral detection in a child without clinical, laboratory or radiographic evidence of bacterial co-infection may support withholding or discontinuing antibiotics. The detection of influenza supports antiviral treatment and isolation. Detection of *M. pneumoniae* or *B. pertussis* can redirect therapy toward a more pathogen-appropriate regimen.

Inpatient interpretation must remain cautious. A viral result should not automatically terminate antibacterial treatment in a toxic child with lobar consolidation, high inflammatory biomarkers, pleural effusion or shock. Conversely, persistent antibiotics after the detection of a clear viral pathogen in a stable child without bacterial evidence may represent a missed stewardship opportunity [36]. The value of testing depends on the result being translated into a management decision consistent with the full clinical picture.

Infection prevention is another major ward-level benefit. Rapid identification of RSV, influenza, adenovirus, SARS-CoV-2, PIV or *B. pertussis* can guide single-room isolation, cohorting and ward-level exposure management. In pediatric hospitals where bed capacity is limited, appropriate cohorting may reduce unnecessary isolation days and improve bed utilization, while early recognition of transmissible pathogens may prevent nosocomial spread.

### 3.3. PICU and Severe Pneumonia: Toward Precision Anti-Infective Management

The PICU is a distinct diagnostic environment. Children admitted to the PICU often have rapidly progressive disease, respiratory failure, mechanical ventilation, prior antibiotic exposure, comorbidities or immunocompromising conditions. In this setting, both delayed appropriate therapy and excessive empirical coverage can be harmful [37]. Delayed coverage of resistant bacteria may worsen outcomes, whereas unnecessary broad-spectrum therapy increases toxicity, selection pressure, fungal overgrowth, microbiome disruption and cost [38].

Top pathogens in PICU respiratory infection should include severe respiratory viruses, typical bacterial pathogens, hospital-acquired organisms and resistance markers. RSV, influenza, adenovirus, SARS-CoV-2 and hMPV remain important viral targets. *S. pneumoniae*, *H. influenzae* and *S. aureus* are relevant in severe community-acquired pneumonia [39,40]. In ventilated or hospital-acquired cases, *P. aeruginosa*, *K. pneumoniae*, *A. baumannii*, *Enterobacterales* and MRSA become higher-priority targets, especially when resistance markers can support early escalation or de-escalation [39,40,41,42] (see Table 3).

Most evidence for rapid pneumonia or HAP/VAP panels in ICU settings is derived from adult or mixed ICU populations rather than pediatric-only cohorts. These studies provide important proof of principle that rapid syndromic PCR may improve early antimicrobial appropriateness, but they should be interpreted as indirect evidence for PICU practice. The INHALE WP3 randomized trial in suspected hospital-acquired or ventilator-associated pneumonia enrolled adults and children in 14 ICUs and showed that rapid ICU-based syndromic PCR improved antibiotic stewardship at 24 h by an absolute 21%, although non-inferiority for 14-day clinical cure was not demonstrated [49]. This distinction is important: the earliest measurable benefit of rapid testing may be the improved appropriateness of antimicrobial exposure, while hard clinical endpoints may require larger pediatric studies and optimized behavior-change interventions [49,50]. Pediatric applicability may differ because of differences in airway sampling, pathogen distribution, colonization patterns, immune development, device exposure and the baseline risk of bacterial co-infection. Therefore, conclusions regarding improved precision anti-infective management or stewardship impact in PICU should be considered hypothesis-generating and require pediatric validation.

### 3.4. Hospital-Acquired Pneumonia, Ventilator-Associated Pneumonia and Outbreak Settings

Hospital-acquired pneumonia (HAP) and ventilator-associated pneumonia (VAP) require a different top-pathogen hierarchy from community-acquired disease [51]. In these scenarios, resistant Gram-negative bacteria and MRSA are more prominent, and rapid resistance information can be clinically actionable [52]. Lower-airway sampling quality, bacterial load, prior antibiotic exposure and colonization must be considered carefully. A positive result from a pneumonia panel may support early targeted therapy, but it must be integrated with clinical signs, radiography, ventilator parameters, inflammatory markers and culture results and follow-up. However, pediatric HAP/VAP data remain limited, and much of the current evidence supporting pneumonia or HAP/VAP panels is extrapolated from adult or mixed ICU studies. Therefore, in children, panel results should be used to support, rather than replace, clinical assessment, the evaluation of lower-airway specimen quality, culture confirmation and antimicrobial stewardship review.

Outbreak and infection-control settings create another reason to prioritize certain pathogens. RSV, influenza, SARS-CoV-2, adenovirus, PIV and *B. pertussis* can affect pediatric wards, neonatal units and PICUs [53]. Rapid testing enables isolation, cohorting, exposure notification, staff and visitor precautions and, when appropriate, post-exposure prophylaxis. Here, the economic value of testing may extend beyond individual patient management: prevention of bed closures, staff exposure, nosocomial infection and ward-level outbreaks may help offset testing costs [54].

These settings illustrate why top-pathogen selection is dynamic [55]. A pathogen may be a priority because it is treatable, transmissible, associated with deterioration, resistant, or relevant to hospital operations (Table 3). A single static respiratory panel cannot fully capture these changing priorities.

### 3.5. Clinical Value Pathways

The clinical value of scenario-driven rapid testing can be summarized through six pathways. First, rapid testing shortens time to etiologic information, particularly when results return before antibiotic, antiviral, isolation or disposition decisions are finalized. Second, it improves antimicrobial appropriateness by supporting either targeted therapy or de-escalation. Third, it may reduce unnecessary antibiotic exposure, but only when bacterial co-infection risk is low and clinicians follow an agreed pathway. Fourth, it enables timely antiviral therapy, especially for influenza. Fifth, it optimizes isolation and cohorting for transmissible pathogens. Sixth, when combined with age, severity, oxygen requirement, biomarkers and host-response signatures, pathogen identity may contribute to risk stratification and PICU decision-making.

These pathways should become the basis of outcome measurement. Diagnostic studies should report not only positivity rates and turnaround time, but also antibiotic initiation, discontinuation and de-escalation; antiviral use; isolation days; length of stay; PICU transfer; repeated testing; total cost; and family-level burden (see Table 4).

## 4. Economic Value, Implementation Strategy and Future Directions

### 4.1. Economic Value Beyond Test Cost

The economic value of rapid top-pathogen testing should not be assessed solely by comparing the price of a test with the price of conventional culture or antigen detection. A more informative framework should consider the relationship among upfront testing and implementation costs, the likelihood that results will change clinical management and the downstream costs that may be avoided when management changes occur. These downstream effects include diagnostic work-up, antimicrobial use, emergency department length of stay, hospital admission, ward length of stay, PICU stay, isolation resources, bed utilization, nosocomial transmission and family burden [61,62].

Direct medical costs include the test itself, platform and laboratory requirements, personnel time, antibiotics, antivirals, radiography, laboratory monitoring, inpatient bed-days, PICU care, mechanical ventilation, isolation and infection-control resources. Indirect family costs are particularly important in pediatrics and include missed work by caregivers, transportation, accommodation, repeated visits, time spent in hospital and psychological stress. Economic evaluations that ignore caregiver burden may underestimate the total value of rapid diagnosis.

Evidence remains heterogeneous. A pediatric economic analysis of influenza testing found that rapid multiplex PCR could be the most effective strategy and may be cost-effective under certain conditions, but results were sensitive to influenza prevalence, antiviral use and test cost [63,64]. In contrast, broad multiplex point-of-care testing in an unselected pediatric ED population did not reduce overall costs [14]. These findings support a conditional view: rapid testing can generate economic value when it is applied to populations with a meaningful probability of actionable pathogens and when results return early enough to change treatment, isolation, admission or discharge decisions. Conversely, when testing is applied broadly to low-risk populations without an expected management change, it may add diagnostic costs without producing measurable downstream savings.

### 4.2. Conditions Required for Cost-Effectiveness

A scenario-driven rapid-testing pathway is most likely to be cost-effective when four conditions are met. First, the tested population should have a sufficiently high pre-test probability of actionable pathogens, which depends on pathogen prevalence, age, season, clinical syndrome, disease severity and care setting. Second, the test should have an acceptable cost relative to the expected downstream savings, and results must return within the relevant clinical decision window [65]. Third, the result should have a realistic probability of changing management, such as antibiotic withholding, discontinuation or de-escalation, earlier antiviral therapy, targeted escalation for resistant bacteria, optimized isolation, avoidance of unnecessary admission or earlier discharge [66]. Fourth, clinicians should be supported by diagnostic and antimicrobial stewardship tools that translate results into action [67].

Under this framework, the major determinants of economic value include pathogen prevalence, assay and implementation cost, turnaround time, probability of management change, reduction in unnecessary antibiotic exposure, admission avoidance, emergency department or hospital length of stay, PICU utilization, isolation days, repeated testing, bed utilization and the prevention of healthcare-associated transmission [63]. Their relative importance differs by setting: in the ED, value depends mainly on whether testing affects antiviral use, antibiot ic prescribing, observation time or disposition [14]; in inpatient wards, value depends more on antibiotic de-escalation, isolation duration and bed utilization; and in PICU, HAP or VAP settings, value depends on whether resistance information supports the earlier optimization of broad-spectrum therapy [68].

The same framework helps distinguish scenarios in which testing is likely to save resources from those in which it mainly adds diagnostic costs. Resource savings are most plausible when testing is used in patients with a high probability of actionable pathogens, when results return before key treatment, isolation, admission or discharge decisions, and when clinicians are prepared to act on the result. Examples include influenza testing during seasonal circulation when antiviral therapy or isolation decisions may change, CAP-focused testing when results support antibiotic optimization, and PICU/HAP/VAP testing when resistance information guides early escalation or de-escalation. By contrast, broad testing is more likely to add diagnostic costs when used in low-risk children, when results return after disposition decisions, when positive results are difficult to interpret, or when clinicians do not change management.

Implementation costs and resource availability should also be considered. Broad multiplex panels, pneumonia/HAP/VAP panels and mNGS require assay costs, platform maintenance, laboratory infrastructure, trained personnel, result interpretation and stewardship support. These requirements may be feasible in high-income healthcare systems or tertiary pediatric centers, but may be difficult to implement in middle-income or lower-resource hospitals with limited access to PCR or mNGS. In such settings, a tiered strategy may be more realistic: targeted testing or small panels for high-volume ED or ward scenarios, and broader panels or mNGS reserved for severe, unexplained, immunocompromised or PICU cases.

Thus, testing may increase diagnostic certainty without generating clinical or economic value if results do not lead to timely management changes. The practical message is that rapid testing becomes cost-effective only when diagnostic information is translated into timely clinical action and when downstream savings or clinical benefits are sufficient to offset the costs of testing and implementation (see Table 5).

### 4.3. Diagnostic Stewardship and Antimicrobial Stewardship

Diagnostic stewardship is essential for maximizing value and preventing overuse. A survey from the Society for Healthcare Epidemiology of America Research Network showed that many institutions perceive multiplex molecular respiratory panels as useful, but also commonly use diagnostic stewardship strategies such as structured order sets, restrictions based on clinician or patient characteristics and structured result communication [69]. These approaches are directly relevant to pediatric respiratory testing.

A pediatric hospital implementation package should include scenario-based order criteria, age- and season-specific test menus, electronic decision support, reflex-testing rules, interpretive reporting, linkage with antimicrobial stewardship review and the regular audit of test utilization [70]. For example, an ED order set could recommend a small top-pathogen panel for infants with bronchiolitis requiring oxygen [44], while a PICU order set could recommend a broader pneumonia panel plus cultures for ventilated patients with suspected HAP/VAP [71]. Reporting should avoid isolated pathogen labels and instead provide interpretive comments such as ‘viral pathogen detected; bacterial co-infection should be assessed based on clinical severity, biomarkers and radiology’ or ‘resistance marker detected; antimicrobial stewardship or infectious disease consultation recommended.’

Antimicrobial stewardship should be integrated from the beginning rather than added after testing is implemented. Without stewardship, positive viral results may not lead to antibiotic discontinuation, bacterial detections may drive unnecessary escalation, and broad panels may increase interpretive ambiguity. This reflects a form of diagnostic inertia: clinicians may continue empirical antibiotics despite viral detection or negative bacterial results when they remain concerned about bacterial co-infection, severe disease, poor sample quality or the discordance between test results and the clinical picture. Therefore, rapid testing should not be expected to change prescribing behavior by itself. The diagnostic pathway must accordingly specify expected actions for positive, negative and multiple-positive results and should be combined with interpretive reporting, antimicrobial stewardship review and clinician education to translate results into appropriate management.

### 4.4. Challenges and Pitfalls

Several pitfalls can limit the value of scenario-driven testing. The first is causality. Detection does not prove that the organism is responsible for disease. This issue is particularly important for rhinovirus, adenovirus, *S. pneumoniae* and *H. influenzae* in upper-airway samples [72,73]. The second is co-detection. Multiple-positive results may reflect mixed infection, sequential infection, colonization or residual shedding, and may require clinical interpretation rather than automatic treatment escalation [74]. The third pitfall is false reassurance from negative results. A negative panel does not exclude infection if sampling quality is poor, the pathogen is outside the panel, disease is localized in the lower airway or antimicrobial exposure has modified pathogen burden. Fourth, broad panels may encourage low-value testing if ordered in children with low pre-test probability or no expected management change [10]. This may lead to overtesting, particularly in high-volume ED settings where rapid tests are easily available and may be used for diagnostic reassurance rather than for a predefined clinical decision. In low-prevalence or low-risk populations, positive results may be false-positive or clinically misleading because of contamination, residual nucleic acid, colonization or low-level detections of uncertain relevance. Poorly targeted testing may also contribute to diagnostic fatigue and increase the burden of interpreting multiple low-actionability or discordant results. In the ED, rapid testing supports workflow only when turnaround time is aligned with triage, isolation, treatment or disposition decisions; otherwise, testing may prolong observation, delay disposition or increase staff and laboratory workload without improving management.

Fifth, local epidemiology matters. RSV seasonality, influenza waves, *M. pneumoniae* epidemics, *pertussis* resurgence, vaccination coverage and resistance ecology differ across regions and years [75]. This local variability also highlights an important limitation of the proposed framework: no universally validated weighting system currently exists for pediatric top-pathogen prioritization. Therefore, the framework should be regarded as a locally adaptable decision aid that requires prospective evaluation and periodic recalibration using local epidemiological, microbiological and stewardship data, rather than as a fixed universal ranking system. Sixth, pediatric PICU-specific evidence remains limited, especially in settings outside North America and Europe [76].

### 4.5. Future Directions: Dynamic Panels, Host Response and Real-World Evidence

Future rapid-testing strategies should move from static respiratory panels toward dynamic, data-driven top-pathogen menus. Pediatric hospitals can use real-world laboratory and clinical data to update pathogen priorities by age group, season, syndrome, ward, severity and resistance pattern [77,78]. Emergency departments may benefit from small, high-actionability panels, whereas PICUs may require broader panels incorporating bacterial pathogens, resistance markers and lower-airway sampling.

The integration of pathogen detection with host-response profiling is a particularly important direction [79]. Pathogen detection identifies the presence of microbial nucleic acid or antigen, whereas host-response biomarkers may help determine whether the detected organism is clinically relevant to the current disease process. This distinction is especially important in children, in whom broad multiplex panels may detect rhinovirus or adenovirus during prolonged shedding, or identify *S. pneumoniae* and *H. influenzae* as upper-airway colonizers rather than true causes of lower-airway infection. Therefore, combining pathogen detection with host-response information may help address one of the major interpretive challenges of broad panels: distinguishing colonization or residual detection from true infection. Host transcriptomic signatures and interferon-response profiles may help distinguish viral, bacterial and mixed-infection patterns, while conventional inflammatory biomarkers such as C-reactive protein, white blood cell count and procalcitonin may help estimate the probability of bacterial co-infection or invasive bacterial disease. However, these markers should not be used as stand-alone diagnostic rules, because their interpretation may be influenced by age, immune development, timing of sampling, comorbidities, prior antimicrobial exposure and disease severity. Their greatest potential lies in integrated algorithms that combine pathogen results, host-response markers, radiology, clinical severity and stewardship guidance. Future pediatric studies should evaluate whether pathogen-plus-host-response algorithms improve the interpretation of positive PCR results, reduce unnecessary antibiotic escalation and improve the recognition of bacterial co-infection compared with pathogen testing alone.

Large pediatric healthcare systems also have an opportunity to generate real-world economic evidence. Prospective studies should compare standard care with scenario-driven top-pathogen pathways in ED and PICU settings. Outcomes should include time to pathogen identification, antibiotic initiation and discontinuation, antiviral use, isolation duration, admission, PICU transfer, length of stay, total hospital cost and family economic burden [35]. Future studies should also evaluate whether pathogen-plus-host-response algorithms improve the interpretation of positive PCR results, reduce unnecessary antibiotic escalation and improve the recognition of bacterial co-infection compared with pathogen testing alone. China and other countries with high pediatric respiratory disease volume can contribute particularly valuable evidence by integrating local pathogen surveillance, clinical pathway redesign and cost-effectiveness analysis (see Table 6).

## 5. Conclusions

Rapid pathogen testing in pediatric respiratory infections should not be judged solely by the number of organisms detected. Its clinical and economic value depends on whether testing is targeted to the clinical scenario, focused on actionable top pathogens and embedded within diagnostic and antimicrobial stewardship pathways. Pediatric ED trials caution that rapid testing alone may not reduce antibiotic use, whereas syndrome-specific testing in pediatric CAP can improve antimicrobial appropriateness. In PICU and HAP/VAP settings, rapid bacterial and resistance detection may support more precise anti-infective management, but pediatric-specific outcome evidence remains limited and conclusions are partly extrapolated from adult or mixed ICU studies.

**Figure 1:**
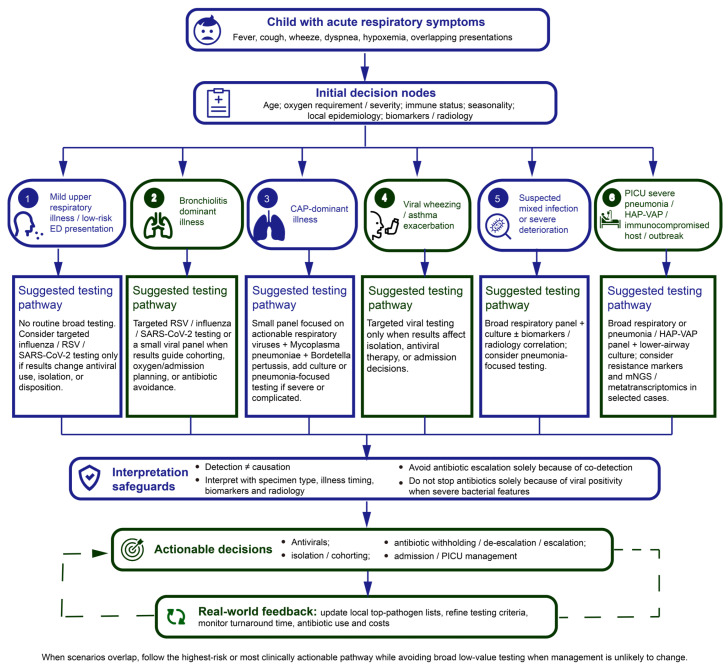
Scenario-driven top-pathogen rapid-testing framework for pediatric respiratory infections.

A scenario-driven top-pathogen framework offers a practical strategy for aligning laboratory innovation with pediatric clinical decision-making. By linking age, syndrome, severity, local epidemiology and expected management actions, this approach may improve diagnostic efficiency, reduce unnecessary antimicrobial exposure, optimize isolation and bed utilization, and lower healthcare and family economic burden. Future research should prioritize real-world pediatric studies that combine rapid testing, stewardship interventions, host-response biomarkers and health-economic evaluation (see Figure 1).

## Figures and Tables

**Table 1:** Operational prioritization matrix for scenario-driven top-pathogen testing in pediatric respiratory infections.

Criterion	Operational Question	High-Priority Indicator	Example
Local prevalence/seasonality	Is the pathogen common in this age group, season or ward?	High local positivity rate or ongoing outbreak/seasonal activity	RSV in infants during winter; influenza during seasonal circulation
Disease severity	Is it associated with hypoxemia, hospitalization, PICU admission or complications?	Associated with severe pneumonia, respiratory failure or death	RSV in young infants; adenovirus/influenza in severe pneumonia
Clinical actionability	Does detection change treatment, admission, isolation or follow-up?	Changes antivirals, antibiotics, cohorting, admission or public-health action	Influenza, *B. pertussis*, *M. pneumoniae*
AMR relevance	Does it influence escalation or de-escalation of antibacterial therapy?	Pathogen or resistance marker changes empirical coverage	MRSA, *P. aeruginosa*, carbapenem-resistant Gram-negative bacteria
Transmission/infection control	Does detection affect isolation, cohorting or outbreak response?	High transmissibility or ward outbreak potential	RSV, influenza, SARS-CoV-2, adenovirus, pertussis
Turnaround-time compatibility	Can the result return before a key decision?	Result available before antibiotics, antivirals, disposition or isolation decision	ED influenza/RSV/SARS-CoV-2 testing
Economic/operational impact	Could testing reduce avoidable costs or resource use?	Potential to reduce antibiotics, isolation days, bed closure, repeated testing or length of stay	Ward RSV cohorting; PICU HAP/VAP resistance-informed testing

**Table 2:** Matching rapid diagnostic platforms to pediatric respiratory clinical questions.

Platform	Core Strength	Main Limitation	Best-Use Pediatric Scenario	References
Rapid antigen test	Fast, inexpensive and operationally simple	Lower sensitivity than nucleic acid amplification; negative results may not exclude infection	ED or outpatient screening for influenza, RSV or SARS-CoV-2 when a positive result changes isolation, antiviral use or disposition	[28]
Singleplex PCR	High analytical performance for one target	Limited coverage when several pathogens are plausible	High suspicion of one actionable pathogen, such as pertussis or influenza during seasonal circulation	[29]
Small multiplex PCR	Balances coverage, speed, cost and interpretability	Requires local design and periodic updating	ED or ward top-pathogen testing for RSV, influenza, SARS-CoV-2, hMPV, adenovirus, *M. pneumoniae* and *B. pertussis*	[29]
Broad respiratory panel	High diagnostic breadth and rapid simultaneous detection	Cost, interpretation burden and risk of low-value testing	Hospitalized, immunocompromised or PICU patients; severe pneumonia; suspected mixed infection; outbreak investigation	[30]
Pneumonia/HAP/VAP panel	Detects bacterial pathogens and selected resistance markers	Most informative when lower-airway disease is likely and specimens are appropriate	PICU severe pneumonia, ventilated patients, suspected HAP/VAP or prior broad-spectrum antibiotic exposure	[25]
mNGS/metatranscriptomics	Unbiased detection; can identify unusual pathogens and support integrated pathogen–host analysis	Cost, turnaround time, contamination and interpretation challenges	Severe unexplained pneumonia, immunocompromised children, negative conventional testing or suspected rare/mixed infection	[31]

**Table 3:** Practical scenario classification for pediatric respiratory rapid testing before etiology is known.

Initial Presentation Before Etiology Is Known	Key Decision Points at Presentation	Suggested Initial Testing Pathway	References
Mild upper respiratory illness or low-risk ED presentation	No hypoxemia, no focal pneumonia signs, no high-risk host factors, and test results are unlikely to change treatment or disposition	Avoid routine broad multiplex testing. Consider targeted influenza, RSV or SARS-CoV-2 testing during seasonal circulation, known exposure or infection-control events if results would affect antiviral use, isolation advice or disposition.	[43]
Bronchiolitis-dominant illness in infants	Young age, wheezing or crackles, seasonal RSV activity, oxygen requirement or admission risk, and no strong evidence of bacterial pneumonia	Use targeted testing or a small viral panel when results would guide cohorting, isolation, oxygen/admission planning or avoidance of unnecessary antibiotics.	[44]
Viral wheezing or asthma exacerbation-dominant illness	Recurrent wheezing or asthma history, viral trigger, response to bronchodilator therapy, no focal consolidation or strong bacterial features	Use targeted viral testing when results would affect isolation, antiviral therapy or admission decisions. Broaden testing only when disease is severe, hospitalization is required or bacterial co-infection is suspected.	[45]
Community-acquired pneumonia-dominant illness	Fever, tachypnea, focal auscultatory findings, radiographic pneumonia, hypoxemia, school-age child or atypical pneumonia features	Use a small respiratory panel focused on actionable respiratory viruses and atypical pathogens. Add bacterial culture or pneumonia-focused testing when disease is severe, complicated or associated with high bacterial co-infection risk.	[11]
Suspected bacterial co-infection or mixed infection	Severe illness, persistent or recurrent fever, high inflammatory biomarkers, lobar consolidation, pleural effusion, shock, clinical deterioration, or discordance between viral detection and clinical severity	Select a broader respiratory panel and add bacterial culture or pneumonia panel when lower-airway disease is likely. Interpret viral positivity together with biomarkers, radiology and clinical severity rather than using it to automatically exclude bacterial infection.	[46]
PICU severe pneumonia or respiratory failure	Respiratory failure, mechanical ventilation, shock, immunocompromised status, prior antibiotic exposure or rapid deterioration	Use broad respiratory testing plus bacterial culture and pneumonia/HAP/VAP panel with bacterial and resistance targets. Consider mNGS or metatranscriptomics when conventional testing is negative, unusual pathogens are suspected or the host is immunocompromised.	[31]
HAP/VAP-dominant scenario	Hospital-onset respiratory symptoms, ventilator-associated deterioration, prior broad-spectrum antibiotic exposure, local resistance problem, or suspected resistant bacterial infection	Use a pneumonia or HAP/VAP panel plus respiratory and blood cultures when bacterial lower-airway infection is suspected and resistance-informed treatment decisions are needed.	[47]
Outbreak or infection-control scenario	Ward cluster, known exposure, seasonal outbreak, high transmissibility concern, or the need for cohorting, exposure notification or prophylaxis	Use targeted testing for transmissible respiratory pathogens such as influenza, RSV, SARS-CoV-2, adenovirus, PIV or *B. pertussis* when results would affect isolation, cohorting, exposure notification or prophylaxis.	[48]

**Table 4:** Scenario-specific top pathogens and expected clinical actions in pediatric respiratory infections.

Clinical Scenario	Priority Top Pathogens	Main Testing Purpose	Recommended Action	Action Not Recommended	Evidence Basis	References
Mild ED respiratory illness	Influenza, RSV, SARS-CoV-2	Fast triage and infection-control decision	Use targeted testing when results may affect antiviral therapy, isolation advice, observation or discharge planning.	Do not use broad multiplex testing routinely in low-risk children when results are unlikely to change management.	Pediatric or indirect evidence	[56]
Infant bronchiolitis	RSV, rhinovirus, hMPV, PIV, influenza	Etiologic clarification and cohorting	Use results to guide cohorting, isolation and supportive-care planning; consider admission risk together with oxygen requirement and clinical severity.	Do not use viral detection alone to justify antibiotics or to automatically exclude bacterial co-infection in severe or atypical cases.	Pediatric guideline/evidence	[57]
Pediatric CAP	Influenza, RSV, adenovirus, *M. pneumoniae*, *B. pertussis*, *S. pneumoniae*	Improve antimicrobial selection	Consider antivirals when indicated, optimize antibiotic selection and target atypical pathogens when supported by clinical features.	Do not interpret upper-airway bacterial detection alone as definitive bacterial pneumonia; avoid unnecessary antibiotics in likely viral pneumonia without bacterial features	Pediatric guideline/evidence	[11]
Persistent or paroxysmal cough	*B. pertussis*, *M. pneumoniae*, respiratory viruses	Targeted therapy and public-health response	Use targeted results to guide macrolide therapy, contact management, school or household guidance and public-health measures.	Do not use broad respiratory panels as a substitute for targeted pertussis or atypical-pathogen testing when the syndrome is specific.	Pediatric guideline/evidence	[58]
PICU severe pneumonia	RSV, influenza, adenovirus, *S. aureus*, *S. pneumoniae,* Gram-negative bacteria	Support precision anti-infective management	Use broader testing with cultures and stewardship review to support escalation, de-escalation, antiviral therapy or mixed-infection assessment.	Do not treat panel results as replacing clinical assessment, lower-airway specimen quality evaluation, biomarkers, radiology or culture confirmation.	Adult/mixed ICU extrapolation	[59]
HAP/VAP	*P. aeruginosa*, *K. pneumoniae*, *A. baumannii*, *Enterobacterales*, MRSA, resistance genes	Resistance-informed treatment	Use pneumonia/HAP/VAP panel results to support early targeted therapy, resistance-informed escalation or narrowing when resistance targets are absent; confirm with cultures.	Do not rely on panel results alone without clinical signs, ventilator parameters, lower-airway sample quality and culture follow-up.	Adult/mixed ICU extrapolation	[60]
Outbreak or ward transmission	RSV, influenza, SARS-CoV-2, adenovirus, PIV, *B. pertussis*	Infection prevention and operational control	Use targeted testing for isolation, cohorting, exposure notification, visitor/staff precautions and prophylaxis when indicated.	Do not perform broad testing without a clear infection-control or public-health consequence.	Pediatric or infection-control evidence	[53]

**Table 5:** Scenarios in which rapid testing is likely to generate value versus mainly adding diagnostic cost.

Likely Value-Generating Testing	Likely Low-Value or Cost-Adding Testing
Influenza-like illness during influenza season when antivirals or isolation decisions may change	Mild URI where results will not change treatment, isolation or disposition
Infant bronchiolitis requiring admission or cohorting	Broad multiplex panel for every outpatient cough
CAP with uncertain need for antibiotics or atypical coverage	Viral panel ordered after antibiotics and admission decisions are finalized
PICU severe pneumonia requiring escalation/de-escalation decisions	Broad panel without culture, biomarkers or stewardship interpretation
HAP/VAP with need for resistance-informed therapy	Upper-airway PCR used alone to define VAP etiology
Ward outbreak or high-risk transmission setting	Repeated testing without a new clinical decision point

**Table 6:** Clinical and economic endpoints for evaluating scenario-driven top-pathogen rapid testing.

Endpoint Domain	Representative Indicators	Interpretive Value
Diagnostic efficiency	Time to result; time to pathogen identification; proportion of results available before treatment/disposition decision	Determines whether testing occurs within the actionable decision window
Antimicrobial management	Antibiotic initiation, discontinuation, de-escalation, escalation, duration; antiviral initiation	Measures translation of diagnostic results into treatment decisions
Clinical utilization	ED length of stay; admission rate; inpatient length of stay; PICU transfer; PICU length of stay; mechanical ventilation duration	Captures resource use and disease trajectory
Infection control	Isolation days; cohorting accuracy; exposure events; ward outbreaks; bed closures	Captures operational and public-health value beyond individual diagnosis
Cost	Test cost; drug cost; imaging/laboratory cost; inpatient cost; PICU cost; isolation cost; total hospital cost	Determines whether downstream savings offset testing costs
Family burden	Caregiver missed work; transportation; accommodation; repeated visits; rehospitalization; caregiving time	Captures pediatric-specific societal and household economic impact

## Data Availability

No new data were created or analyzed in this study.

## Abbreviations

The following abbreviations are used in this manuscript:

*A. baumannii*	*Acinetobacter baumannii*
*B. pertussis*	*Bordetella pertussis*
CAP	Community-acquired pneumonia
ED	Emergency department
*H. influenzae*	*Haemophilus influenzae*
HAP	Hospital-acquired pneumonia
hMPV	Human metapneumovirus
ICU	Intensive care unit
*K. pneumoniae*	*Klebsiella pneumoniae*
*M. pneumoniae*	*Mycoplasma pneumoniae*
MRSA	Methicillin-resistant *Staphylococcus aureus*
*P. aeruginosa*	*Pseudomonas aeruginosa*
PCR	Polymerase chain reaction
PICU	Pediatric intensive care unit
PIV	Parainfluenza virus
RSV	Respiratory syncytial virus
*S. aureus*	*Staphylococcus aureus*
SARS-CoV-2	Severe acute respiratory syndrome coronavirus 2
*S. pneumoniae*	*Streptococcus pneumoniae*
VAP	Ventilator-associated pneumonia
WHO	World Health Organization
